# Interferon-γ: The Jekyll and Hyde of Malaria

**DOI:** 10.1371/journal.ppat.1005118

**Published:** 2015-10-01

**Authors:** Thayer King, Tracey Lamb

**Affiliations:** Department of Pediatrics, Emory University School of Medicine, Atlanta, Georgia, United States of America; University of Wisconsin Medical School, UNITED STATES

## Introduction

Interferon gamma (IFN-γ) is a key mediator of inflammatory immune responses induced primarily by interleukin-12 (IL-12). IFN-γ secretion by both innate and adaptive immune cells is essential for control of intracellular pathogens and tumors, yet aberrant production of IFN-γ contributes to autoimmunity and inflammation in certain disease settings. These divergent roles are well illustrated in the context of malaria, a disease caused by infection with protozoan parasites of the genus *Plasmodium*. IFN-γ is a central cytokine in controlling *Plasmodium* infection in both the liver and blood stages of the parasite life cycle, but it can also exacerbate the severity of malarial disease depending on the temporal and spatial production of IFN-γ. Here, we review the types of immune cells that produce IFN-γ during malaria and discuss the IFN-γ-induced effector mechanisms that can aid in killing *Plasmodium* parasites but also contribute to the pathogenesis of malaria.

## Which Immune Cells Produce IFN-γ during Malaria?

*Plasmodium* infection induces IFN-γ production from a variety of innate and adaptive immune cell subsets at different stages of the life cycle. Studies in mice have demonstrated that natural killer (NK) cells are one of the earliest sources of IFN-γ during the liver stage [1], as well as blood stage [2], of malaria. For example, C57BL/6J mice depleted of NK cells and infected with a nonlethal *Plasmodium yoelli* strain showed a 58% abrogation of IFN-γ production at 24 hours postinfection [2]. Human NK cells have also been shown to rapidly produce IFN-γ upon incubation with *Plasmodium falciparum*-infected red blood cells (iRBCs) in vitro [3]. Bridging innate and adaptive immunity, both natural killer T (NKT) cells and γδ T cells can contribute to IFN-γ production during *Plasmodium* infection. Studies suggest a significant proportion (50%) of γδ T cells from humans infected with *P*. *falciparum* secrete IFN-γ [4], while NKT cells in mice secrete IFN-γ in response to sporozoites and liver stage parasites [5]. While there is likely significant redundancy in IFN-γ production from leukocytes in response to both liver stage and blood stage *Plasmodium* parasites, studies using IFN-γ eYFP reporter mice infected with *P*. *berghei* ANKA suggest that NK cells contribute greater to IFN-γ production than both NKT and γδ T cells at early time points postinfection, and the production of IFN-γ from NKT and γδ T cells remains fairly stable over time [6].

Once an adaptive immune response is initiated, both CD4^+^ and CD8^+^ T cells become a major source of IFN-γ in response to both liver stage [7] and blood stage malaria. The finding that both CD4^+^ [8] and CD8^+^ [9] T cells isolated from *Plasmodium*-infected humans produce IFN-γ is also observed in many mouse models of malaria. Secretion of IFN-γ by both CD4^+^ and CD8^+^ T cells increases around day seven postinfection with blood stage *P*. *berghei* ANKA in both the spleen and brain [6]. While IFN-γ is the canonical cytokine that has been used to define CD4^+^ T cells as Th1 cells, it has been widely observed that Th1 cells can simultaneously produce other inflammatory cytokines including IL-2, TNF-α, and IL-17 during an adaptive immune response. A subset of IFN-γ/IL-10 double-producing CD4^+^ T cells have been observed in humans infected with *Plasmodium* [8,10], and mouse models of malaria suggest that IFN-γ/IL-10 double-producing cells are an important source of IL-10 that limit immunopathogenesis of malaria [11] at the cost of inhibiting control of the infection [12].

## What Evidence Suggests That IFN-γ Is Protective during Malaria?

There have been several correlations between IFN-γ levels in the periphery and protection against severe malaria in humans. The protective capacity of IFN-γ in malaria appears to be, in part, related to the timing of IFN-γ production with the early appearance of IFN-γ after infection in humans correlated with protection against the development of clinical symptoms of malaria in some studies [13]. However, study conclusions are often complicated by factors that include differing patterns of *Plasmodium* transmission between study sites or varying levels of pathogen coinfection giving rise to conflicting data. Experiments in mice also suggest that early IFN-γ production is protective against experimental cerebral malaria (ECM), and peripheral levels of IFN-γ can drop just before the onset of ECM [14] with a similar phenomenon potentially occurring in humans [15]. This introduces a time-dependent sampling variable that can pose problems when attempting to establish a correlation between disease severity and peripheral IFN-γ levels. Nevertheless, in a study where human volunteers were infected over time with several low doses of *Plasmodium* iRBCs and treated to clear the infection, protection from a challenge infection was positively correlated with numbers of circulating IFN-γ-producing CD4^+^ T cells [16]. The natural resistance of the Fulani tribe in Mali to *Plasmodium* infection has also been correlated with elevated levels of IFN-γ [17], suggesting a protective role for IFN-γ against malaria.

Similar to human malaria, IFN-γ also appears to play a protective role against blood stage *Plasmodium* infection in mice. Mice lacking IFN-γ experience higher and more prolonged blood stage parasitemia compared to IFN-γ-sufficient mice when infected with the rodent parasites *P*. *yoelii yoelii* or *P*. *chabaudi adami* [18]. Additionally, a separate study found that IFN-γ levels were markedly higher 24 hours post blood stage infection in mice infected with nonlethal strains of *P*. *chabaudi* or *P*. *yoelli* when compared to mice infected with lethal strains of *P*. *yoelli* or *P*. *berghei* [2] emphasizing the potential benefits of IFN-γ to disease control in mice.

The prevalence of IFN-γ-producing CD4^+^ and CD8^+^ T cells has been associated with a greater likelihood of uncomplicated malaria [8], as well as reduced severe malarial anemia [9] in humans. However, experiments in mice have demonstrated that IFN-γ production by NK cells, NKT cells, and γδ T cells can also play a major role in the control of *Plasmodium* infection. NKT cells have been shown to inhibit parasite growth within hepatocytes in a partially IFN-γ-dependent manner [5]. Also, despite the prominent role of CD4^+^ and CD8^+^ T cells in contributing to serum IFN-γ levels during malaria, γδ T cells in mice are able to control liver stage *Plasmodium* infection in the absence of αβ T cells [19], demonstrating that γδ T cells are an important source of IFN-γ with respect to parasite control.

## What Immune Effector Mechanisms Responsible for Controlling *Plasmodium* Infection Are Activated by IFN-γ?

**Fig 1:**
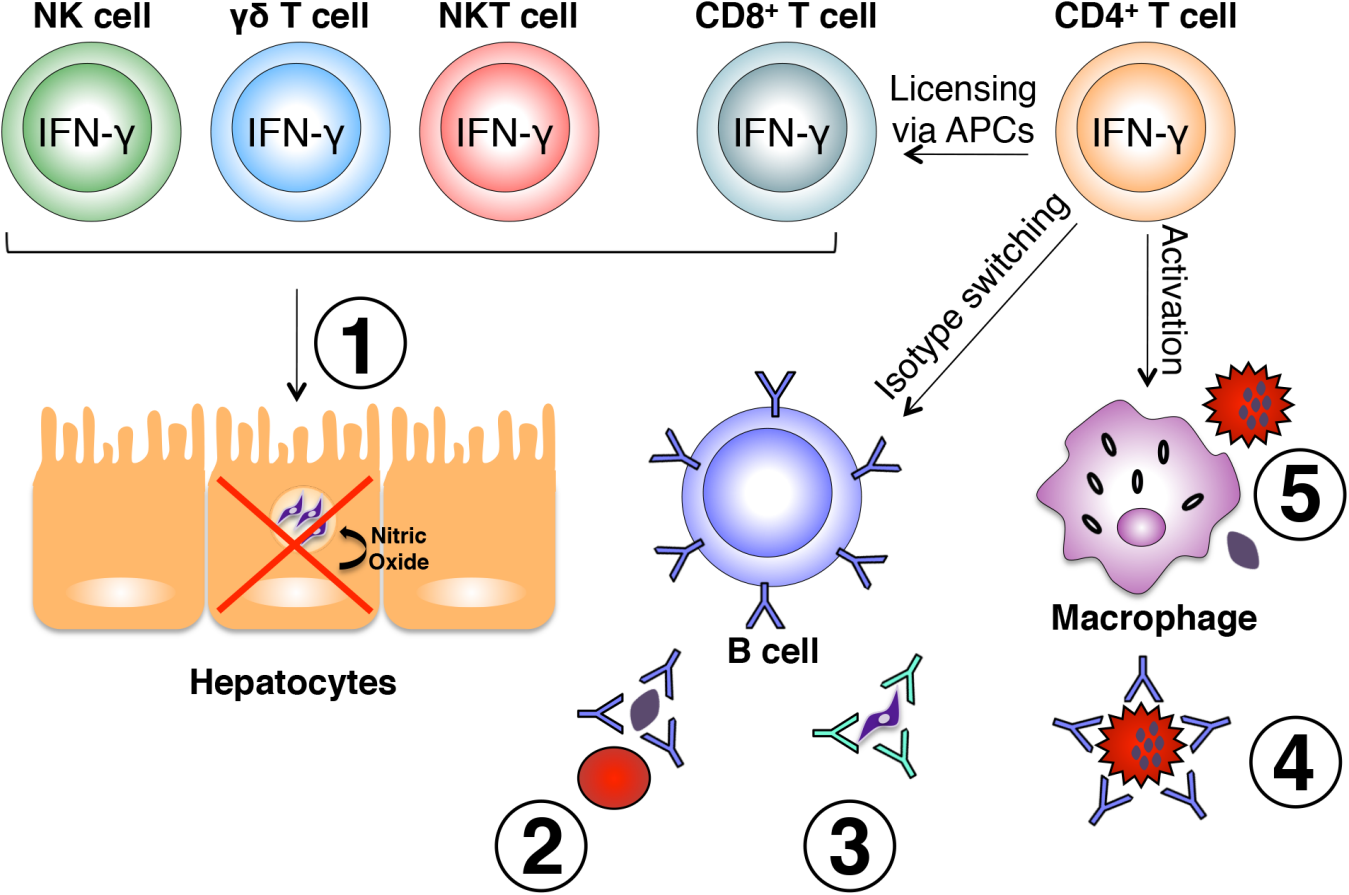
Effector mechanisms induced by IFN-γ during malaria. Various immune cell subsets produce IFN-γ in response to *Plasmodium* infection. NK, γδ, and NKT cells are largely responsible for early production of IFN-γ in response to liver and blood stages of the parasite and play a role in early control of parasite growth. IFN-γ-producing CD8^+^ T cells have also been shown to limit intrahepatic parasite growth through an IFN-γ-inducible, nitric oxide-dependent mechanism (**1**). Once an adaptive immune response is initiated, IFN-γ produced by CD4^+^ T cells optimally activates CD8^+^ T cells, B cells, and macrophages. IFN-γ influences isotype switching in B cells leading to production of cytophilic antibodies capable of binding free parasites and blocking red blood cell invasion (**2**), mediating parasite clearance through opsonization (**3**), and binding the surface of infected red blood cells promoting antibody-dependent phagocytosis (**4**). Production of IFN-γ from CD4^+^ T cells also optimally activates macrophages to phagocytose infected red blood cells and free parasites (**5**). All of these mechanisms are important for optimal control of parasite growth during *Plasmodium* infection.

IFN-γ secreted by CD4^+^ Th1 cells is critical for optimal activation of CD8^+^ T cells, B cells, and macrophages, all of which perform vital roles in the control of *Plasmodium* infection (Fig 1). The primary immune effector mechanisms by which IFN-γ can influence destruction of *Plasmodium*-infected cells include increasing the cytotoxic potential of CD8^+^ T cells, inducing production of cytophilic antibodies by B cells and enhancing phagocytic abilities of immune cells such as macrophages. It should be noted that the latter two functions are not mutually exclusive.

IFN-γ exerts its effects on immune cells that express the IFNGR1/2 cell surface receptor, and signaling through this receptor results in activation of transcription factors such as IRF1, STAT1, JAK2, IRF9, CIITA [20], and T-bet [21]. This leads to expression of a number of proteins such as nitric oxide synthase and FcγRI (CD64, a high-affinity Fc receptor) [20], as well as induction of B cell class-switching to the IgG2a antibody isotype [21]. As a result, the aforementioned events can lead to enhanced phagocytosis and destruction of intracellular pathogens.

Regarding liver stage parasite development in mice, CD8^+^ T cells induced by a DNA vaccination encoding the gene for a *P*. *yoelli* liver stage antigen were shown to be absolutely essential for protection of mice from a *P*. *yoelli* sporozoite challenge infection [22]. This protection was entirely dependent on IFN-γ production from *Plasmodium*-specific CD8^+^ T cells as well as IFN-γ-inducible nitric oxide synthase production from *Plasmodium*-infected hepatocytes [22] leading to direct intracellular parasite killing.

Although CD8^+^ T cell responses have also been implicated in control of blood stage *Plasmodium* infection in an IFN-γ-dependent manner [23], the mechanism by which this could occur remains unclear since infected red blood cells do not express the major histocompatibility complex class I (MHC-I) which is required for CD8^+^ T cell recognition. On the contrary, antibodies are known to be key effector molecules in *Plasmodium* infection and perform many well-characterized functions important for parasite control and clearance such as blocking parasite reinvasion, parasite opsonization, and targeting of parasites for phagocytosis. IFN-γ impacts the antibody response and isotypes of malaria-specific antibodies produced, which is evident in IFN-γ^-/-^ mice that produce significantly less parasite-specific IgM, IgG3, and cytophilic IgG2a than wild-type mice [24]. Thus, the role of IFN-γ in the isotype switching of B cells to a cytophilic antibody isotype such as IgG2a increases the opsonic potential of antibodies.

IFN-γ is a potent activator of macrophages and can increase canonical macrophage activities such as phagocytosis and the production of both proinflammatory cytokines and reactive oxygen intermediates. In mice, IFN-γ has been shown to enhance phagocytosis of *P*. *chabaudi* AS iRBCs and free merozoites by peritoneal macrophages with mice lacking IFN-γ exhibiting lower levels of parasite phagocytosis than wild-type mice [25]. The phagocytosis-enhancing role of IFN-γ has been further demonstrated in vitro as macrophages isolated from C57BL/6J mice treated with IFN-γ exhibit enhanced phagocytosis of iRBCs, an effect inhibited by IL-10 treatment [25]. Since phagocytosis of iRBCs can occur in an antibody-dependent or antibody-independent manner, the role of IFN-γ on B cells and phagocytes is likely synergistic leading to increased phagocytosis of opsonized parasites and parasite products.

## How Does IFN-γ Cause Pathology during Malaria?

The pathology associated with malaria is caused by the blood stages of *Plasmodium* infection and, in particular, immune responses targeting iRBCs sequestered in various organs. In susceptible C57BL/6J mice infected with *P*. *berghei* ANKA, which develop experimental cerebral malaria (ECM), the IFN-γ-induced immune response against iRBCs sequestered in the brain and lung is required for pathogenesis of infection [6]. Furthermore, IFN-γ^-/-^ mice that are resistant to ECM show decreased parasite and leukocyte accumulation in the brain [26]. This finding has been attributed to robust IFN-γ-induced expression of canonical adhesion molecules such as ICAM-1 [26], as well as CD4^+^ T cell IFN-γ-induced expression of the chemokines CXCL9 and CXCL10 that recruit IFN-γ-producing CD8^+^ T cells to the brain during ECM [6]. These pathogenic T cells induce cerebral pathology [6] most likely through perforin- and granzyme-dependent disruption of the blood-brain barrier upon recognition of malaria-derived peptides presented in the context of MHC-I on brain endothelial cells [27]. Thus, while IFN-γ is necessary for the recruitment of CD8^+^ T cells to the brain during ECM [28], other effector molecules produced by activated CD8^+^ T cells such as perforin and granzyme B are the critical mediators of pathology [29].

## Perspectives

Studies in both humans and mice suggest a fine line between IFN-γ-associated protection versus immunopathology in the immune response to malaria in which a threshold level is needed for optimal control of parasitemia, yet aberrant expression can lead to pathology and the complications of severe malaria. It is tempting to explore boosting the IFN-γ response during malaria therapeutically, given the associations between IFN-γ and improved disease outcome. For example, phase IIa clinical trials for the RTS,S pre-erythrocytic malaria vaccine candidate showed a correlation between prolonged CD4^+^ and CD8^+^ T cell IFN-γ responses against a malaria-specific protein and protection upon challenge infection in human volunteers [30]. In mice, the loss of protective immunity against *P*. *chabaudi* is associated with a decrease in memory CD4^+^ Th1 cells after parasite clearance along with a concomitant decrease in IFN-γ production from splenocytes [31]. Splenic IFN-γ has been shown to be required for optimal priming of effector and effector memory T cells by splenic innate cells [32], demonstrating the importance of this cytokine in maintenance of optimal immunity. However, it is clear that the dynamic roles of IFN-γ during malaria are complex, and more work is needed to understand the delicate balance of IFN-γ necessary for achieving optimal protection while minimizing pathology.
